# Supplementary material for: Psychological distress among health service providers during COVID-19 pandemic in Nepal
Source: PLoS One. 2021 Feb 10;16(2):e0246784. doi: 10.1371/journal.pone.0246784 (PMC7875377; doi:10.1371/journal.pone.0246784)
Supplement: S1 File — (DOCX) [file pone.0246784.s001.docx]

**S1 File.**

**PSYCHOLOGICAL DISTRESS AMONG HEALTH SERVICE PROVIDERS DURING COVID-19 PANDEMIC IN NEPAL**

**1. Questionnaire used in Google form**

[INVITATION TO PARTICIPATE IN AN ONLINE SURVEY TO ASSESS PSYCHOLOGICAL DISTRESS DURING COVID-19 PANDEMIC HEALTH SERVICE PROVIDERS IN NEPAL]

INTRODUCTION TO RESEARCH

COVID-19 pandemic has caused serious threats to people’s physical health and lives. It may also trigger a wide range of psychological responses such as panic, anxiety, and depression. It is important to know that these responses are normal in situations like a pandemic. However, if you are experiencing worry, fear, sadness, hopelessness and other similar feelings a lot of the time for more than two weeks, it may indicate psychological distress. This study is the first nationwide large-scale survey to assess psychological distress among health service providers of Nepal who are in very high number, battling against this pandemic as the first line workers.

PURPOSE OF RESEARCH

The main purpose of this study is to measure the prevalence and severity of this psychological distress, gauge the current mental health burden on h, and therefore provide a concrete basis for tailoring and implementing relevant mental health intervention policies to cope with this challenge efficiently and effectively.

PARTICIPATION IN RESEARCH, BENEFITS, AND RISK

Your participation in this research is entirely voluntary. You may refuse to take part in the study or you may withdraw yourself from participation in the research at any time without penalty. The outcome of the study will help to establish a crisis prevention and intervention system to reduce psychological distress and prevent further mental health problems during a pandemic. Information obtained from this research will benefit the individuals, researchers, institution and community for the advancement of knowledge and future practice. Engagement in this study is minimal risk if you follow the instruction given to you. If you would like to know the results of the study, we will notify you. However, if you have urgent problems please consult a qualified medical professional.

HOW LONG IT TAKES

The procedure involves filling an online survey that will take approximately 6-8 minutes.

CONFEDEDIALITY

Your information will be kept confidential by the investigators and will not be made public unless disclosure is required by law. By signing this consent form, you will authorize the review of records, analysis and use of the data arising from this research.

The research was approved by the Ethical Committee of Chitwan Medical College (CMC), Bharatpur, Chitwan.

PAYMENT AND COMPENSATION

You do not have to pay for participating in this study. Similarly, no payment is available to you for participating in this study

- - Required

1. Email address *


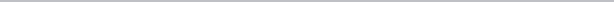


AGREEMENT TO PARTICIPATE

Your participation is completely voluntary, and you can withdraw at any time. To take this survey, you must be

1. At least 18 years old and above and
2. Health Service Providers (Doctors, Dentist,Nurses, Lab technicians and assistant, radiology technician and assistant,public heath worker etc) in any institution at Nepal

If you meet the above criteria and would like to take the survey, click the button "YES" below to start the survey

By clicking on "yes" I here with confirm that:

1. I voluntarily agree to take part in this research and to provide all necessary information to the investigators as requested.

2. I may at any time choose to withdraw from this research without giving any reason.

3. I agree to hold them harmless from any harm or loss that may be incurred by me due to my participation in the research. * *

1. DO YOU AGREE TO PARTICIPATE IN THE SURVEY * *Mark only one oval.*


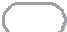
 No


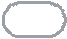
 Yes

Thank you for agreeing to participate in this study

1. Name:
2. Date of participaton


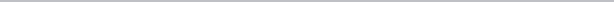

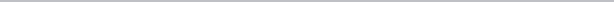


*Example: January 7, 2019*

1. Your State of residence

* *Mark only one oval.*


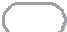
 1


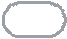
 2


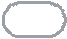
 3 (Bagmati pradesh)


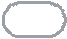
 4 (Gandaki Pradesh)


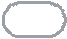
 5


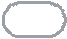
 6 (karnali pradesh)


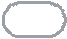
 7 (sudurpaschim pradesh)

1. Nationality :

**Mark only one oval.*


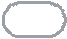
 Nepali


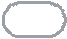
 Non nepali

1. Gender

**Mark only one oval.*


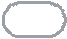
 Male


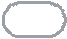
 Female

1. Marital status

**Mark only one oval.*


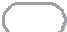
 Married


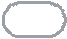
 unmarried

Widow/divorced

1. Ethnicity

**Mark only one oval.*


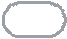
 Brahmin


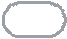
 Chhetri


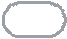
 Janajati


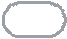
 Dalits


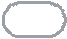
 Yadav


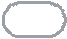
 others

1. Religion

**Mark only one oval.*


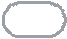
 Hindu


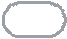
 Buddhist


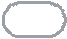
 Muslims


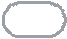
 Christains


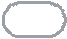
 others

1. Age in years:


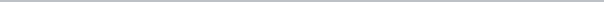


1. Education

**Mark only one oval.*


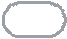
 Diploma Level


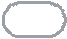
 Bachelors Level


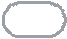
 Master Level

1. Employment

**Mark only one oval.*


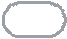
 Doctor


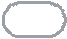
 Dentist


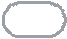
 Nursing staff


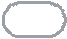
 Lab staff


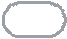
 Radiology staff


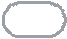
 Public health worker


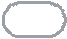
 Others

1. Currently working institution Status

* *Mark only one oval.*


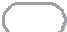
 Government institution


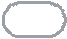
 private institution


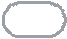
INGO's

1. type of institution currently working

* *Mark only one oval.*


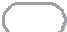
 Health post


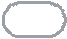
 PHC


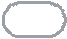
 Hospital


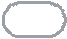
 Tertiary care centre

others

1. how many weeks do you work per month

* *Mark only one oval.*


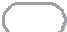
 1


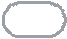
 2


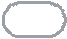
 3

- 4

1. working hours per week

* *Mark only one oval.*


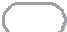
 Less than 40 hours


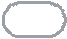
 40 hours

More than 40 hours

1. Protective equipment used by you

* *Mark only one oval.*


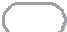
 Mask only


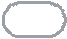
 mask and gloves


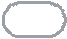
 mask, gloves and face shield

complete PPE set

1. Are you receiving allowance for working in this pandemic?

**Mark only one oval.*


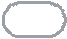
 Yes


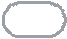
 No


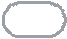
 may be, But not yet

Questions 1-24 asking your feeling on current pandemic COVID 19.Please select the most appropriate answer from the options below.

Choose only best option (scroring mark Equivalent: 1=Never/2=occasionally /3= Sometimes/4.=Often/5= Always)

Question 1: Compared to usual, I feel more nervous and anxious.

* *Mark only one oval.*


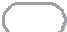
 Never


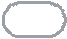
 Occasionally


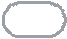
 Sometimes


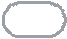
 Often


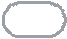
 Always

Question 2: I feel insecure and bought a lot of masks, medications, sanitizers, gloves and/or other home supplies.

**Mark only one oval.*


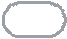
 Never


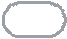
 Occasionally


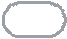
 Sometimes


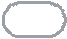
 Often


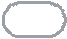
 Always

Question 3: I can’t stop myself from imagining myself or my family being infected and feel terrified and anxious about it.

**Mark only one oval.*


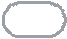
 Never


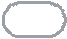
 Occasionally


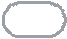
 Sometimes


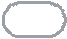
 Often


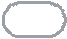
 Always

Question 4: I feel helpless no matter what I do.

* *Mark only one oval.*


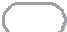
 Never


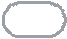
 Occasionally


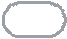
 Sometimes


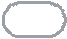
 Often


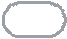
 Always

Question 5: I feel sympathetic to COVID-19 patients and their families.

* *Mark only one oval.*


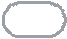
 Never


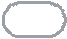
 Occasionally


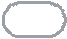
 Sometimes


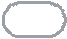
 Often


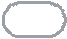
 Always

Question 6: I feel helpless and angry about people around me, governors, and media.

* *Mark only one oval.*


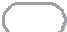
 Never


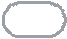
 Occasionally


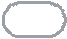
 Sometimes


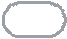
 Often


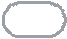
 Always

Question 7: I am losing faith in the people around me.

* *Mark only one oval.*


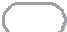
 Never


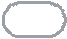
 Occasionally


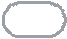
 Sometimes


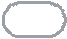
 Often


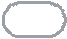
 Always

Question 8: I collect information about COVID-19 all day. Even if it’s not necessary, I can’t stop myself.

**Mark only one oval.*


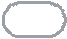
 Never


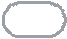
 Occasionally


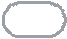
 Sometimes


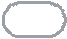
 Often


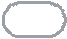
 Always

Question 9: I will believe the COVID-19 information from all sources without any evaluation.

* *Mark only one oval.*


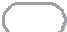
 Never


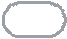
 Occasionally

Sometimes

Often

Always

Question 10: I would rather believe in negative news about COVID-19 and be skeptical about the good news.

**Mark only one oval.*

Never

Occasionally

Sometimes

Often

Always

Question 11: I am constantly sharing news about COVID-19 (mostly negative news). * *Mark only one oval.*

Never

Occasionally

Sometimes

Often

Always

Question 12: I avoid watching COVID-19 news since I am too scared to do so.

* *Mark only one oval.*

Never

Occasionally

Sometimes

Often

Always

Question 13: I am more irritable and have frequent conflicts with my family * *Mark only one oval.*

Never

Occasionally

Sometimes

Often

Always

Question 14: I feel tired and sometimes even exhausted. * *Mark only one oval.*

Never

Occasionally

Sometimes

Often

Always

Question 15: When feelings anxious, my reactions are becoming sluggish * *Mark only one oval.*

Never

Occasionally

Sometimes

Often

Always

Question 16: I find it hard to concentrate.

* *Mark only one oval.*

Never

Occasionally

Sometimes

Often

Always

Question 17: I find it hard to make any decisions * *Mark only one oval.*

Never

Occasionally

Sometimes

Often

Always

Question 18: During this COVID-19 period, I often feel dizzy or have back pain and chest distress. * *Mark only one oval.*

Never

Occasionally

Sometimes

Often

Always

Question 19: During this COVID-19 period, I often feel stomach pain, bloating, and other stomach discomforts. *

*Mark only one oval.*

Never

Occasionally

Sometimes

Often

Always

Question 20: I feel uncomfortable when communicating with others. * *Mark only one oval.*

Never

Occasionally

Sometimes

Often

Always

Question 21 - I talked with my family members very rarely. * *Mark only one oval.*

Never

Occasionally

Sometimes

Often

Always

Question 22: I have frequent awakening at night due to my dream about myself or my family being infected by COVID-19. *

*Mark only one oval.*

Never

Occasionally

Sometimes

Often

Always

Question 23: I have changes in my eating habits * *Mark only one oval.*

Never

Occasionally

Sometimes

Often

Always

Question 24: I have constipation or frequent urination. * *Mark only one oval.*

Never

Occasionally

Sometimes

Often

Always

| Interpretation of score: (0-28 is Normal).....(>28 and ≤ 51 indicates mild to moderate distress; ) ..... | (≥52 |
| --- | --- |
| Indicates severe distress.) |  |

There are various volunteer organization / Individual in Nepal who provide psychosocial support for your mental health problem. If you feel you are in distress please seek counseling support. Below is contact of one volunteer organization in Nepal.

Counseling Psychology Nepal

NB: you can contact one of our researcher and psychiatrist

([khagendrakafle@gmail.com](mailto:khagendrakafle@gmail.com))

# 2. Table 1. Predictors of CPDI through binary logistics regression

| Socio-demographic Variables | | Exp(B) | 95% CI for Exp(B) | | p-value |
| --- | --- | --- | --- | --- | --- |
| Age | <30 | .000 | 0.000 |  | 1.000 |
|  | 30-45 | .000 | 0.000 |  | 1.000 |
|  | >45 ® |  |  |  | .947 |
| Gender | Female | 1.548 | .783 | 3.058 | .209 |
|  | Male ® |  |  |  |  |
| Religion | Non-Hinduism | .435 | .154 | 1.234 | .118 |
|  | Hinduism ® |  |  |  |  |
| Education | Diploma | 1.755 | .602 | 5.120 | .303 |
|  | Bachelor or master® |  |  |  |  |
| Employment | Doctor | 1.149 | .543 | 2.427 | .717 |
|  | Nurse | 2.225 | .862 | 5.747 | .099 |
|  | Other HCW ® |  |  |  | .242 |
| Current Job | Government | .697 | .372 | 1.307 | .261 |
|  | Non-Government ® |  |  |  |  |
| Institute category | Hospitals or higher center | .974 | .443 | 2.137 | .947 |
|  | PHC, Health post or others ® |  |  |  |  |
| Work in weeks per month | Less than 4 weeks per month | 1.748 | .792 | 3.858 | .167 |
|  | 4 weeks per month ® |  |  |  |  |
| Duty HRS per week | Less than 40 hrs | .645 | .276 | 1.503 | .309 |
|  | More than or equal 40 hrs ® |  |  |  |  |
| Use of PPE | Complete | .930 | .432 | 2.000 | .852 |
|  | Incomplete ® |  |  |  |  |
| Extra allowance | May be or Yes | .878 | .485 | 1.587 | .667 |
|  | No ® |  |  |  |  |
| Marital status | Married | .000 | 0.000 |  | .999 |
|  | Unmarried | .000 | 0.000 |  | .999 |
|  | Widowed or divorced ® |  |  |  | .687 |
| Nationality | Non-Nepali | 4.599 | .148 | 142.444 | .384 |
|  | Nepali ® |  |  |  |  |
| Ethnicity | Others | 1.616 | .857 | 3.047 | .138 |
|  | Brahmin and chettri ® |  |  |  |  |
| Residence | Province 1 (Briatnagar as territorial capital) | .254 | .011 | 5.804 | .391 |
|  | Province 2 (Janakpur as territorial capital) | 1.854 | .097 | 35.571 | .682 |
|  | Province 3 (Bagmati) | .992 | .074 | 13.376 | .995 |
|  | Province 4 (Gandaki) | .605 | .040 | 9.229 | .718 |
|  | Province 5 (Butwal as territorial capital) | 1.282 | .088 | 18.727 | .856 |
|  | Province 6 (Karnali) | 1.402 | .078 | 25.158 | .818 |
|  | Province 7 (Sudurpaschim) ® |  |  |  | .535 |
| ®Reference Category, Significant at 5% level of significance | | | | | |
