# Supplementary material for: Psychological distress among health service providers during COVID-19 pandemic in Nepal
Source: PLoS One. 2021 Feb 10;16(2):e0246784. doi: 10.1371/journal.pone.0246784 (PMC7875377; doi:10.1371/journal.pone.0246784)
Supplement: Attachment — Submitted filename: Rebuttal letter.docx [file pone.0246784.s002.docx]

Joerg Heber,

Editor in Chief, PLOS ONE

Dear Dr. Heber,

Thank you for your interest in our manuscript: PONE-D-20-34279, Psychological Distress among Health Service Providers during COVID-19 Pandemic in Nepal. We are grateful to the editors for allowing us to revise our manuscript. We responded to the reviewer’s comments and concerns, and believe our manuscript is clearer and of greater quality as a result.

Below we repeat each of the comments from the referees in bold italics followed by our responses in plain text with yellow highlights.

Journal requirements:

When submitting your revision, we need you to address these additional requirements.

***1. Please ensure that your manuscript meets PLOS ONE's style requirements, including those for file naming. The PLOS ONE style templates can be found at***

[***https://journals.plos.org/plosone/s/file?id=wjVg/PLOSOne_formatting_sample_main_body.pdf***](https://journals.plos.org/plosone/s/file?id=wjVg/PLOSOne_formatting_sample_main_body.pdf) ***and***

[***https://journals.plos.org/plosone/s/file?id=ba62/PLOSOne_formatting_sample_title_authors_affiliations.pdf***](https://journals.plos.org/plosone/s/file?id=ba62/PLOSOne_formatting_sample_title_authors_affiliations.pdf)

Reply: We have amended in manuscript text.

***2. The text in Figure 1 is hard to read. Please increase the size of the font.***

 Reply: We have amended in manuscript text.

Reviewers' comments:

Reviewer's Responses to Questions

**Comments to the Author**

1. Is the manuscript technically sound, and do the data support the conclusions?
The manuscript must describe a technically sound piece of scientific research with data that supports the conclusions. Experiments must have been conducted rigorously, with appropriate controls, replication, and sample sizes. The conclusions must be drawn appropriately based on the data presented.

Reviewer #1: Partly

Reviewer #2: Yes

2. Has the statistical analysis been performed appropriately and rigorously?

Reviewer #1: I Don't Know

Reviewer #2: Yes

3. Have the authors made all data underlying the findings in their manuscript fully available?

The [PLOS Data policy](http://www.plosone.org/static/policies.action#sharing) requires authors to make all data underlying the findings described in their manuscript fully available without restriction, with rare exception (please refer to the Data Availability Statement in the manuscript PDF file). The data should be provided as part of the manuscript or its supporting information, or deposited to a public repository. For example, in addition to summary statistics, the data points behind means, medians and variance measures should be available. If there are restrictions on publicly sharing data—e.g. participant privacy or use of data from a third party—those must be specified.

Reviewer #1: Yes

Reviewer #2: Yes

4. Is the manuscript presented in an intelligible fashion and written in standard English?

PLOS ONE does not copyedit accepted manuscripts, so the language in submitted articles must be clear, correct, and unambiguous. Any typographical or grammatical errors should be corrected at revision, so please note any specific errors here.

Reviewer #1: Yes

Reviewer #2: Yes

5. Review Comments to the Author

Please use the space provided to explain your answers to the questions above. You may also include additional comments for the author, including concerns about dual publication, research ethics, or publication ethics. (Please upload your review as an attachment if it exceeds 20,000 characters)

***Reviewer #1: The study raises several major comments and concerns.
1. The measurements of the prevalence of peritraumatic distress related to COVID-19 may be influenced by the timing of the assessment with respect to the temporal evolution of the pandemic. Specifically, the study was conducted in October 2020, i.e., several months after the first outbreak. There is no mention of the potential changes and/or temporal evolution of psychological distress over time and how it could have been influenced the main findings.***

***Do coping mechanisms develop over time or does the burden increase as the pandemic
progresses?***

Reply: Distress severity is contrasted in discussion section. In similar survey in Nepalese residents using CDPI in early phase of pandemic showed distress in only 11%. While present survey showed significantly higher number of participants having distress, could be being this survey was only among health professional who has risk of contracting the infection and next may be due to evolution of pandemic.

***2. Another critical issue is that the setting of the study is unclear, i.e., “hospitals”, “higher
centers”, “health posts” are generic denominations. It is obvious that COVID-19-related
psychological distress is clearly influenced by the fact that healthcare workers have to deal (or not) to patients with COVID-19. Was the study specifically focusing on workers who had to care for this patient group or not? Importantly, the severity of COVID-19 is highly variable (from asymptomatic cases to those in need of ICU admission). Thus, it is
conceivable that people who have to care to ICU cases are more scared and distressed than those working in a general ward where patients with less severe disease are admitted.***

Reply: This is detailed in methods sections in revision. Available data about institution and type of job is presented in table 1 of result section.

***3. How is it possible to claim that the pandemic has heightened the psychological distress amongst health care service providers? While this is quite expected, the use of CPDI is focused on COVID-19-related distress and broader implications (i.e., “the psychological distress”) are unwarranted and not grounded in the study results.***

Reply: Result of similar study regarding psychological distress in Nepalese community is contrasted in discussion. In prior study, may be due to early phase of pandemic distress level was relatively low in Nepal comparing with other countries, which were already in mid-later phase of pandemic. In present study, we found significantly higher proportion of health professionals with distress suggesting towards its relation with development of pandemic.

***4. The CPDI was developed in China; was it validated in the Nepalese population? Are
psychometric properties of the Nepalese version satisfactory?***

Reply: CPDI is widely under use due to its COVID-19 specific nature. In several studies carried out in Nepal and other countries are using it due to its specific nature. In our case we checked internal consistency of 24 CPDI variables using Cronbach's α. Its internal reliability was found to be 0.905 indicating high internal consistency of the scale.

***5. Most participants were very young. Does working experience mitigate the psychological distress elicited by COVID-19?***

Reply: We have explained in limitation section in discussion.

***Minor points***

***1. Avoid mentioning statistical software in the “Abstract” section.***

Reply: We have amended in manuscript text.

***2. “Gender” is a psychosocial construct; consider “sex” instead. Please also replace “female” and “male” with “women” and “men”, respectively.***

Reply: We have amended in manuscript text.

***Reviewer #2: The study is well written and it focuses on an important aspect of the pandemic which has affected entire health care systems. However, there are several issues that have to be clarified. First of all, the vast majority of the population is less than 30 years old and this may not represent the overall population within the health care system. Second, the institute categories are not well specified especially when considering that the specific conditions in which employees work can affect their psychological distress regardless of the pandemic. Moreover, it's not clear whether all the participants worked directly with COVID-19 patients or not or how this distress level compares to pre-pandemic surveys of healthcare workers.***

Reply: Amended, These issues discussed in method section and limitation section.

Thank you for your consideration. We look forward to hearing from you.

Sincerely,

Dhan Bahadur Shrestha

Mangalbare Hospital, Morang, Nepal
